# Improved Visualization of Cartilage Canals Using Quantitative Susceptibility Mapping

**DOI:** 10.1371/journal.pone.0132167

**Published:** 2015-07-13

**Authors:** Mikko J. Nissi, Ferenc Tóth, Luning Wang, Cathy S. Carlson, Jutta M. Ellermann

**Affiliations:** 1 Center for Magnetic Resonance Research, Department of Radiology, University of Minnesota, Minneapolis, MN, United States of America; 2 Department of Orthopaedic Surgery, University of Minnesota, Minneapolis, MN, United States of America; 3 Research Group of Medical Imaging, Physics and Technology, Faculty of Medicine, University of Oulu, Oulu, Finland; 4 Medical Research Center Oulu, Oulu University Hospital and University of Oulu, Oulu, Finland; 5 Department of Applied Physics, University of Eastern Finland, Kuopio, Finland; 6 Department of Veterinary Population Medicine, College of Veterinary Medicine, University of Minnesota, St. Paul, MN, United States of America; Kyungpook National University School of Medicine, REPUBLIC OF KOREA

## Abstract

**Purpose:**

Cartilage canal vessels are critical to the normal function of epiphyseal (growth) cartilage and damage to these vessels is demonstrated or suspected in several important developmental orthopaedic diseases. High-resolution, three-dimensional (3-D) visualization of cartilage canals has recently been demonstrated using susceptibility weighted imaging (SWI). In the present study, a quantitative susceptibility mapping (QSM) approach is evaluated for 3-D visualization of the cartilage canals. It is hypothesized that QSM post-processing improves visualization of the cartilage canals by resolving artifacts present in the standard SWI post-processing while retaining sensitivity to the cartilage canals.

**Methods:**

Ex vivo distal femoral specimens from 3- and 8-week-old piglets and a 1-month-old human cadaver were scanned at 9.4 T with a 3-D gradient recalled echo sequence suitable for SWI and QSM post-processing. The human specimen and the stifle joint of a live, 3-week-old piglet also were scanned at 7.0 T. Datasets were processed using the standard SWI method and truncated k-space division QSM approach. To compare the post-processing methods, minimum/maximum intensity projections and 3-D reconstructions of the processed datasets were generated and evaluated.

**Results:**

Cartilage canals were successfully visualized using both SWI and QSM approaches. The artifactual splitting of the cartilage canals that occurs due to the dipolar phase, which was present in the SWI post-processed data, was eliminated by the QSM approach. Thus, orientation-independent visualization and better localization of the cartilage canals was achieved with the QSM approach. Combination of GRE with a mask based on QSM data further improved visualization.

**Conclusions:**

Improved and artifact-free 3-D visualization of the cartilage canals was demonstrated by QSM processing of the data, especially by utilizing susceptibility data as an enhancing mask. Utilizing tissue-inherent contrast, this method allows noninvasive assessment of the vasculature in the epiphyseal cartilage in the developing skeleton and potentially increases the opportunity to diagnose disease of this tissue in the preclinical stages, when treatment likely will have increased efficacy.

## Introduction

Susceptibility-weighted imaging (SWI) is an MRI technique that utilizes subtle differences in magnetic susceptibility values between tissues to generate contrast [1–3]. SWI has been primarily used for imaging the brain, including anatomical features [3–5], the venous vasculature [1, 2], areas of hemorrhage and other brain lesions [6, 7], and quantification of iron content [8], areas of calcification [9], and oxygen saturation [10]. Typical SWI approaches rely on high-pass filtering of the phase and subsequent generation and application of a phase mask to the magnitude data [11]. Potential drawbacks of the SWI technique, however, are that it is qualitative and suffers from artifacts due to the dipolar nature of phase accumulation between substances of different magnetic susceptibility [12, 13].

Quantitative susceptibility mapping (QSM) is an approach that attempts to calculate the underlying susceptibility distribution from the phase data [10, 13–16]. The non-uniform susceptibility distribution generates phase changes, from which the susceptibility can be derived by solving an ill-posed inverse problem [13–15, 17]. As the susceptibility distribution is revealed, the result is quantitative as opposed to the qualitative SWI data. Furthermore, the boundaries between susceptibility differences are better defined in the actual susceptibility maps than in the SWI data.

Recently, the utilization of SWI for 3-D visualization of cartilage canal vasculature in the epiphyseal cartilage in the developing skeleton was introduced [12, 18] and its successful application was demonstrated [19]. The vasculature in the epiphyseal cartilage is confined to *cartilage canals*, structures composed of arteries, veins and capillaries embedded in a connective tissue matrix [20]. The diameter of the canals has been reported to range from 0.2 to 0.6 mm, with the confined vessels ranging from 0.01 to 0.16 mm in diameter in young piglets [12, 21]. While the imaging of cartilage canals was demonstrated using SWI with a detection limit roughly scaling with the imaging resolution (canals of approximately 100 μm were detected at 9.4 T at 100 μm isotropic resolution), this method was not free of artifacts [12]. Vessel splitting artifacts, apparent in the SWI data in planes parallel to *B*_0_, resulting from the dipolar phase pattern, hamper three-dimensional assessment and analysis.

The purpose of this study was to explore the application of quantitative susceptibility mapping for the post-processing of cartilage canal data acquired for SWI. We hypothesized that QSM post-processing would allow resolution of the splitting artifact present in the SWI post-processed data and thus improve visualization of cartilage canals in arbitrary 2-D planes or in 3-D reconstructions. To test our hypothesis, cadaveric human and porcine distal femoral specimens were imaged *ex vivo* at 9.4 T and the resulting images were post processed using SWI and QSM. The utility of QSM at a lower field strength and lower resolution was evaluated using data obtained from the same human specimen scanned at 7.0 T. To further evaluate the potential of the approach, a piglet was scanned *in vivo* at 7.0 T and the resulting images were analyzed similarly.

## Methods

### Specimens

A knee joint from a 1-month-old male human cadaver was obtained from Allosource (Allosource, Centennial, CO, USA) and distal femora from 3- and 8-week-old porcine cadavers were randomly selected from animals presented to the Minnesota Veterinary Diagnostic Laboratory for diagnostic necropsy. A 3-week-old piglet for *in vivo* scanning was supplied by the University of Minnesota Research Animal Resources. (Some of the SWI data for the porcine specimens have been previously reported [12, 19], and were re-analyzed in the present study). All animal procedures were approved by the institutional animal care and use committee of the University of Minnesota (protocol: 1111A06521). Specimens were stored at -22°C and thawed at room temperature prior to scanning.

### MR imaging

Imaging of the specimens was conducted at 9.4 T, using an Agilent scanner equipped with VnmrJ software version 3.1 and a quadrature volume transceiver coil (Millipede, Varian NMR Systems, Palo Alto, CA, USA). The specimens were immersed in perfluoropolyether to ensure a proton-free and susceptibility matched background and oriented with the femoral shafts approximately along *B*_0_. Data were acquired using a 3-D gradient recalled echo (GRE) sequence with an isotropic resolution of approximately 100 μm, adjusting the field of view (FOV) and matrix size to fit individual specimens. The scan times varied from 43 minutes to 98 minutes depending on the size of the FOV and, thus, the imaging matrix. For all of the acquisitions, the repetition time (TR) was set to 40 ms and the echo time (TE) was set to 14–15 ms. Acquisition bandwidth was kept to the minimum permitted for the chosen TE, generally around 43 Hz / pixel. The flip angle was set to 15 degrees [12].

The sample obtained from the 1-month-old human cadaver was also imaged at 7.0 T (Magnetom 7T, Siemens, Erlangen, Germany) using an 8-channel transmit/receive [adult size] knee coil (Virtumed, LLC Minneapolis, MN, USA) driven by a *B*_1_ shimming unit (CPC, Hauppauge, NY, USA) with eight 1 kW amplifiers. *B*_1_^+^ shimming was applied in a manually defined region of interest (ROI) in order to maximize flip angle homogeneity as described previously [12, 22, 23]. The data were acquired using a 3-D GRE sequence with TR = 45 ms, TE = 2.46, 4.54, 7.1, 11.2, 17.21 and 29.06 ms with bandwidth = 685, 566, 395, 200, 150 and 60 Hz / pixel, respectively, for each echo time. Variable bandwidth within the same sequence was used to gain both short echo time and high-signal long echo time images in a single shot scan. An isotropic resolution of 320 μm was achieved. For SWI and QSM, the processing was done using the data with the longest echo time; for comparison, unprocessed GRE images acquired with the longest echo time were also assessed.

*In vivo* imaging of the 3-week-old piglet was conducted at 7.0 T as well. The imaging procedure was identical to the *ex vivo* human specimen including *B*_1_^+^ shimming. A single-echo 3-D GRE sequence was used with parameters TR = 27 ms, TE = 15 ms, bandwidth = 90 Hz / pixel with an isotropic resolution of 250 μm. The SWI data of the *in vivo* pig were previously published; further details on the acquisition can be found in reference [12]. For all the 7.0 T acquisitions, the data from individual receive coils was combined using the on-line adaptive combine mode provided by the scanner software; phase and magnitude data output from the scanner were converted to k-space data for further processing.

#### Post-processing

Susceptibility weighted imaging. SWI-post processing of the 3-D GRE data was done according to earlier reports [11, 12, 24]. Briefly, 1) the phase data were high-pass filtered by homodyne filtering using the central half or the central quarter of the k-space in each dimension for the ex vivo and in vivo specimens, respectively [11], 2) a phase mask was created, and 3) the phase mask was applied to the magnitude data (multiplied) four times in all cases [11, 12].

#### Quantitative susceptibility mapping

A 3-D segmentation of the region of interest was done, generating a binary mask for the epiphyseal cartilage, as required by the QSM processing and later steps of the SWI visualization. For specimens scanned at 9.4 T, which were immersed in perfluoropolyether and had a clean ^1^H-signal free background, the segmentation was generated by first applying 3-D smoothing using a 7 x 7 x 7 box convolution kernel to the data, then thresholding using the value $\bar{x}-4\sigma$, where $\bar{x}$ was the average intensity in a manually defined coarse ROI within the area of interest (within epiphyseal cartilage) and σ was the standard deviation of the signal in the same ROI. The mask generated by thresholding was then eroded once (or twice if necessary) using a six-connected 3-D structuring element to increase distance from edges. The final mask was then used in the further post-processing steps. The 7.0 T scans of the 1-month-old cadaveric specimen (immersed in saline) and the 3-week-old *in vivo* piglet were manually segmented and then the manual segmentation mask was fine-tuned by similar 3-D smoothing and erosion steps.

The QSM post-processing of the 3-D GRE data was done using the truncated k-space division as presented by Shmueli et al [13]. Prior to k-space filtering, unwrapping of the phase was first done using Laplacian unwrapping, applying the code provided by Bilgic *et al*, as supplementary material [15], followed by SHARP filtering (using the same code) to remove the background field [14, 15]. The kernel size was set to 9 x 9 x 9, resulting in filter length of approximately 0.9 mm for the 9.4 T data and in 2.3 mm (*in vivo* pig) and 2.9 mm (*ex vivo* human) for the 7.0 T data. The truncation value chosen for SHARP processing was 0.25 for *ex vivo* human at 9.4 T and 0.5 for the *ex vivo* pigs at 9.4 T and for all of the 7.0 T acquisitions. These values were established based on visual inspection of the resulting background-corrected phases, resulting in a markedly larger value than 0.05 used by Bilgic et al [15] and Schweser et al [14]. For the truncated k-space deconvolution filtering [13], a k-space filter kernel 1/(1/3 –k_z_^2^/K^2^) was used. The effect of the kernel truncation value on the resulting susceptibility distribution of the cartilage canals and on their visual appearance was investigated and, finally, truncation of the filter to the minimum absolute values (i.e. to 3/2), was chosen for visualizations to reduce artifacts.

#### Image data post-processing

The final SWI and QSM datasets were masked to remove background and bone signals prior to further processing. For assessment of cartilage canal susceptibility values, an ROI restricted to the canals was generated and histograms as well as averages of the susceptibility values within the ROI were calculated. An ROI consisting of only cartilage canals was created by thresholding the susceptibility data based on a small initial manual ROI to generate an intermediate ROI, which was then manually corrected to exclude non-cartilage canal areas. For visual comparison between the methods, minimum/maximum intensity projections (mIPs or MIPs) through different imaging planes were generated in Matlab (MATLAB R2012b, The MathWorks, Natick, MA, USA) utilizing Aedes and plugins written in house (http://aedes.uef.fi). Similar projections of the unprocessed GRE data were also generated. In addition to the GRE, SWI, and QSM datasets, “enhanced SWI”, QSM-WI datasets of the specimens were generated by turning the quantitative susceptibility maps into positive masks as in standard SWI processing. That is, the susceptibility values smaller than (mean-2*SD) of the cartilage canals were set to 1, while larger values were linearly scaled between 1 and 0 (see for example [25] and [11] on generation of phase mask or two alternative ways of generating QSM-WI [26, 27]). The generated QSM-mask was then applied to the magnitude image four times as in standard SWI processing. Finally, 3-D volume renderings of the masked 3-D datasets were generated in Osirix (Osirix v.5.6 64-bit, http://www.osirix-viewer.com/) [28] to illustrate the differences in visualization by the SWI and QSM processing methods.

## Results

**Fig 1:**
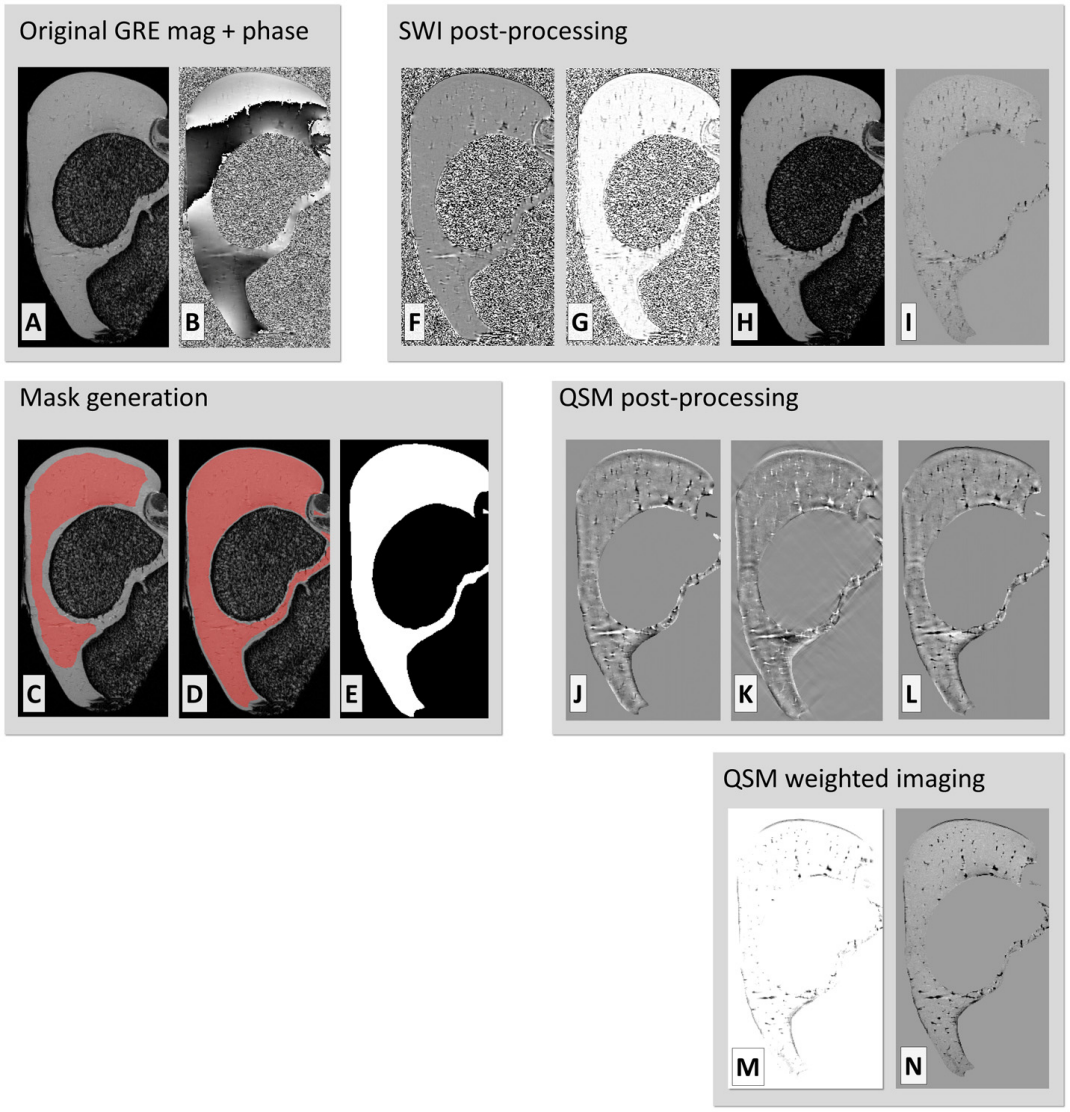
Main pre- and post-processing steps for SWI, QSM and QSM-WI. Main pre- and post-processing steps depicted for a single slice (in a plane parallel to *B*_0_) from the distal femur of a 3-week-old pig at 9.4 T. Original GRE magnitude (A) and phase (B). Generation of segmentation mask was initiated with a single-slice manual ROI (C), which was extended to the entire 3-D volume automatically (D), generating a segmentation mask for further processing (E). In SWI post-processing, high-pass filtering of the phase was first done using homodyne filtering (F). The phase was converted to a negative phase mask (G) and the SWI data was generated by applying the phase mask to the original magnitude data (H). Finally the segmentation mask was also applied to the SWI data for further visualizations (I). For QSM post-processing, the phase was first processed using Laplacian and SHARP filtering (J) and, in turn, converted to a quantitative susceptibility map with k-space inversion (K) and masked with the segmentation and contrast-inverted to match the appearance of SWI (L). Finally, the susceptibility map was converted into an enhancing mask (M) and finally applied to the magnitude data to generate a QSM-WI dataset (N).

To generate the mask for subsequent post-processing, an initial manual ROI was placed on a single, manually chosen slice in the 3-D dataset, exemplified in the distal femur of a 3-week-old piglet at 9.4 T (Fig 1A–1C) The ROI was extended to the entire 3-D volume by filtering and thresholding as described in the methods, and then used as a segmentation mask in the further processing steps (Fig 1D and 1E). After the SWI post-processing (Fig 1F–1H), the segmentation mask was applied to the data for further visualizations (Fig 1I). The quantitative susceptibility map was calculated by first unwrapping the phase and removing the background fields (Fig 1J), followed by k-space filtering to produce the susceptibility map (Fig 1K). The QSM data were masked and the contrast was inverted for further visualizations (Fig 1L). Finally, an enhancing mask based on susceptibility data was generated (Fig 1M) and applied four times to the magnitude data to generate a QSM-WI dataset (Fig 1N).

**Fig 2:**
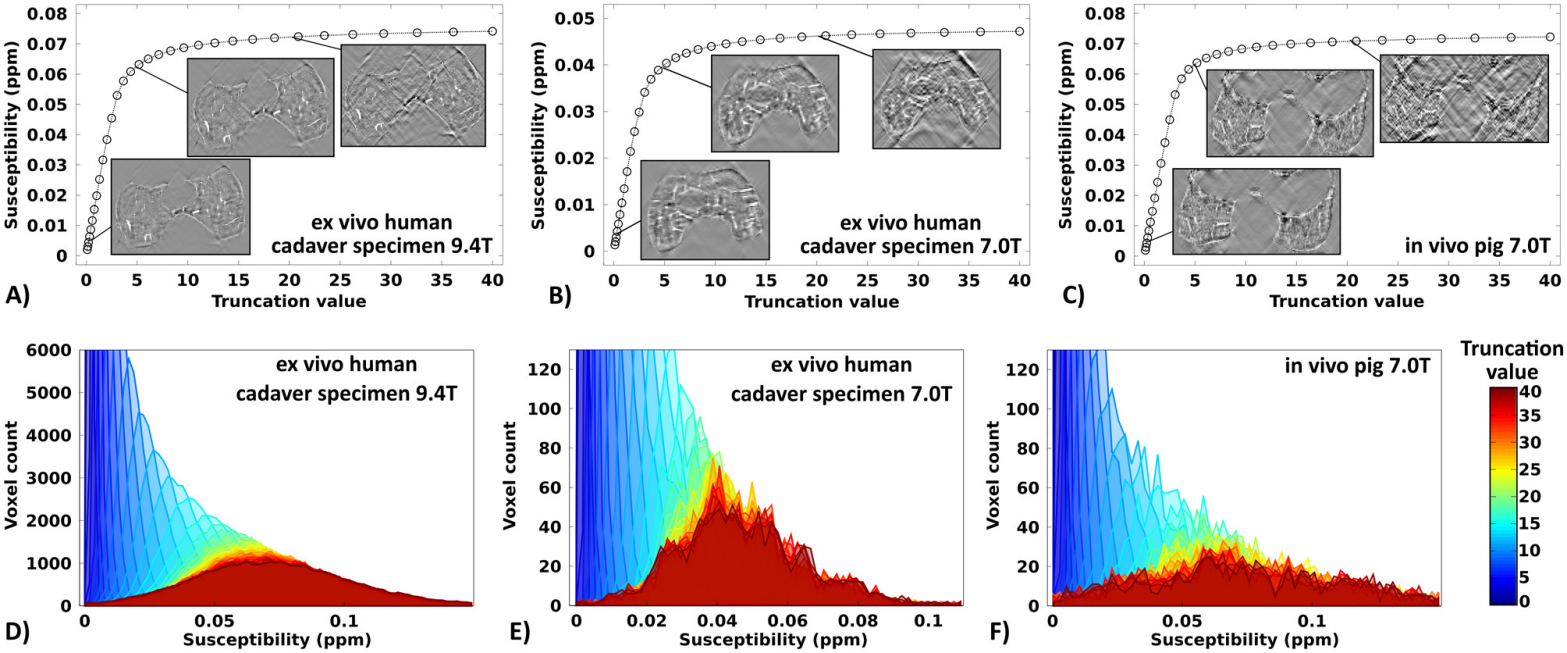
Quantitative susceptibility values of the cartilage canals. Relative susceptibility values of the cartilage canals with respect to the surrounding tissue in a 1-month-old human cadaveric distal femur scanned at 9.4 T (A) and at 7.0 T (B), and in a 3-week-old piglet scanned at 7.0 T *in vivo* (C) as a function of the truncation factor used in the k-space dipole inversion. Inset images in A-C depict single slices from the quantitative susceptibility maps at truncation factor values of 0.5, 5 and 20 at an intensity scale normalized with the intensity of the cartilage canals to facilitate visual comparison of the streaking artifacts. The second row shows the susceptibility histograms acquired for the corresponding cartilage canal ROIs for the respective specimens as a function of the truncation factor (D-F).

The effect of the k-space filter truncation on the calculated relative susceptibility of the cartilage canals (with respect to the surrounding tissue) was studied in the human specimen scanned at 9.4 T and 7.0 T and in the 3-week-old pig scanned *in vivo* at 7.0 T. In all cases, the average susceptibility of the cartilage canals depended on the thresholding value, with the susceptibility increasing as the thresholding was relaxed (Fig 2A–2C). In all cases, the susceptibility values stabilized after the truncation value exceeded approximately 5–10. The spread of the susceptibility values with increasing truncation value was evident in the histograms of the ROIs (Fig 2D–2F). The visual appearance of the susceptibility maps was evaluated at different truncation factor values; an increasing amount of streaking artifacts was observed as the truncation was relaxed (Fig 2A–2C, small inserts). Conversely, as the truncation was heavier, the streaking artifacts were reduced. Below the lowest absolute value of the inverted filter kernel, i.e. below 1.5, no further improvement in artifact reduction was observed and thus 1.5 was chosen as the truncation value for further visualization.

**Fig 3:**
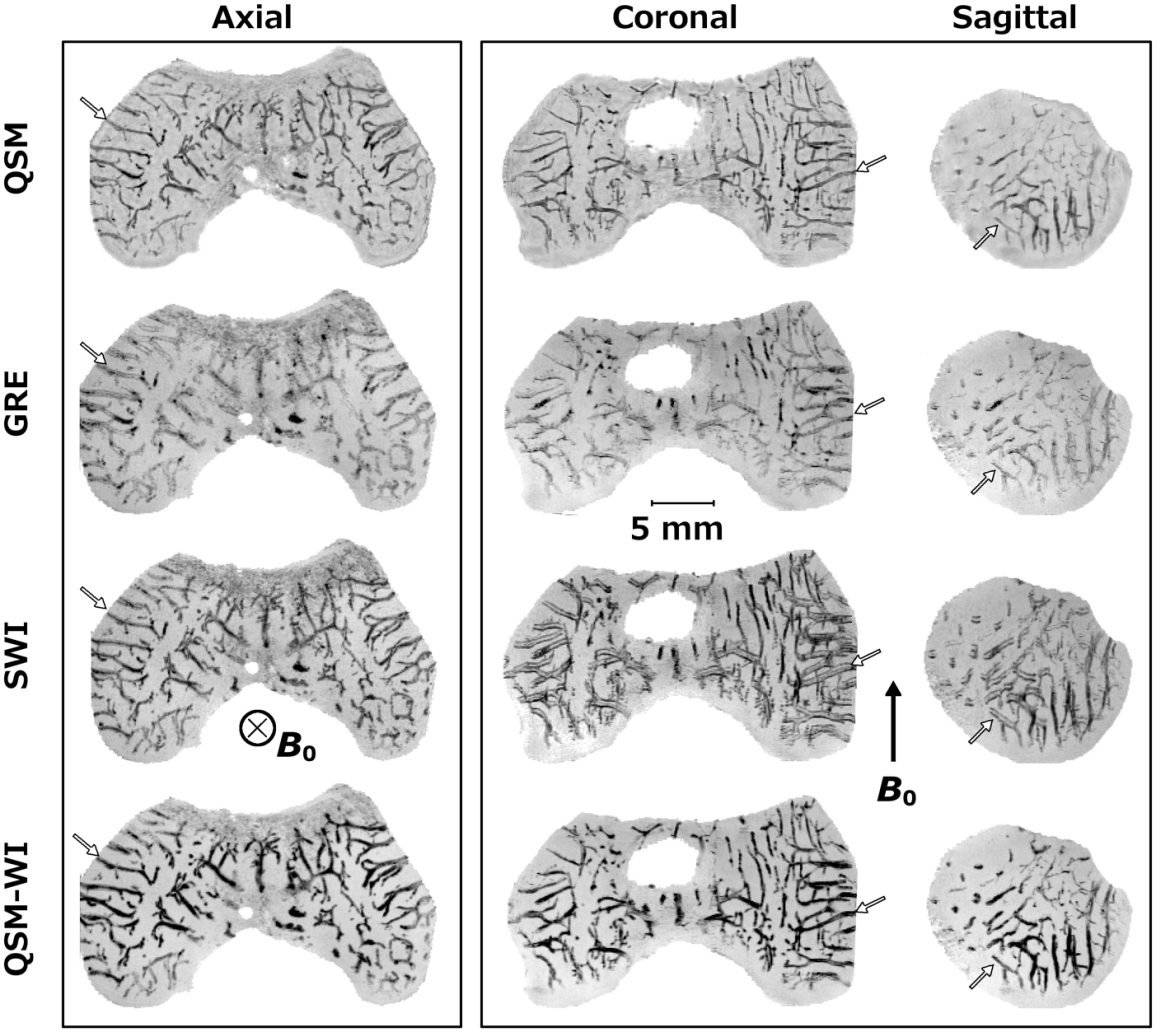
Comparison of QSM, plain GRE, SWI and QSM-WI at 9.4 T. Comparison of QSM, SWI and QSM-WI post-processing as well as unprocessed GRE for the visualization of cartilage canals in a 1-month-old human cadaveric distal femur in 3 mm-thick minimum intensity projections in the main imaging planes with respect to the scanner geometry at 9.4 T (TE = 15.05 ms and bandwidth = 37 Hz/pixel). The first pane shows the axial view, perpendicular to *B*_0_: both truncated k-space QSM (QSM) and QSM-weighted imaging (QSM-WI) results appeared nearly identical to the SWI result. The plain GRE appeared similar, but lacked some of the detail. The second pane shows coronal and sagittal views, parallel to the *B*_0_ field. Both QSM visualizations demonstrate the vasculature without artifacts whereas, in the SWI data, the splitting of the vessels along the *B*_0_ direction is noted. The plain GRE appeared similar to QSM and also did not show artifacts, but clearly lacked the definition seen with QSM. White arrows point to several matching vessels to aid comparison. The QSM contrast (first row) was inverted to match the contrast of the SWI data.

In the plane perpendicular to *B*_0_, with artifacts masked in the SWI data, there was nearly exact correspondence among the QSM, GRE, SWI and QSM-WI mIPs of the distal femur from the 1-month-old human cadaver (Fig 3, first pane). The GRE data showed the least definition while QSM-WI had the highest definition of cartilage canals. In the planes parallel to *B*_0_, (i.e., coronal and sagittal planes), clear visualization of the vasculature was obtained with QSM and QSM-WI post-processing using the truncated k-space filtering (Fig 3, second pane). The un-processed GRE also depicted the vessels, although again with less definition than the other methods, while the SWI visualizations in these planes demonstrated a significant “splitting” artifact, most apparent for vessels running along the imaging plane, perpendicular to *B*_0_ (Fig 3, second pane). (Note that the naming convention of axial, coronal, and sagittal here refers to the axes of the scanner, which closely resemble the anatomic planes of the specimen.)

**Fig 4:**
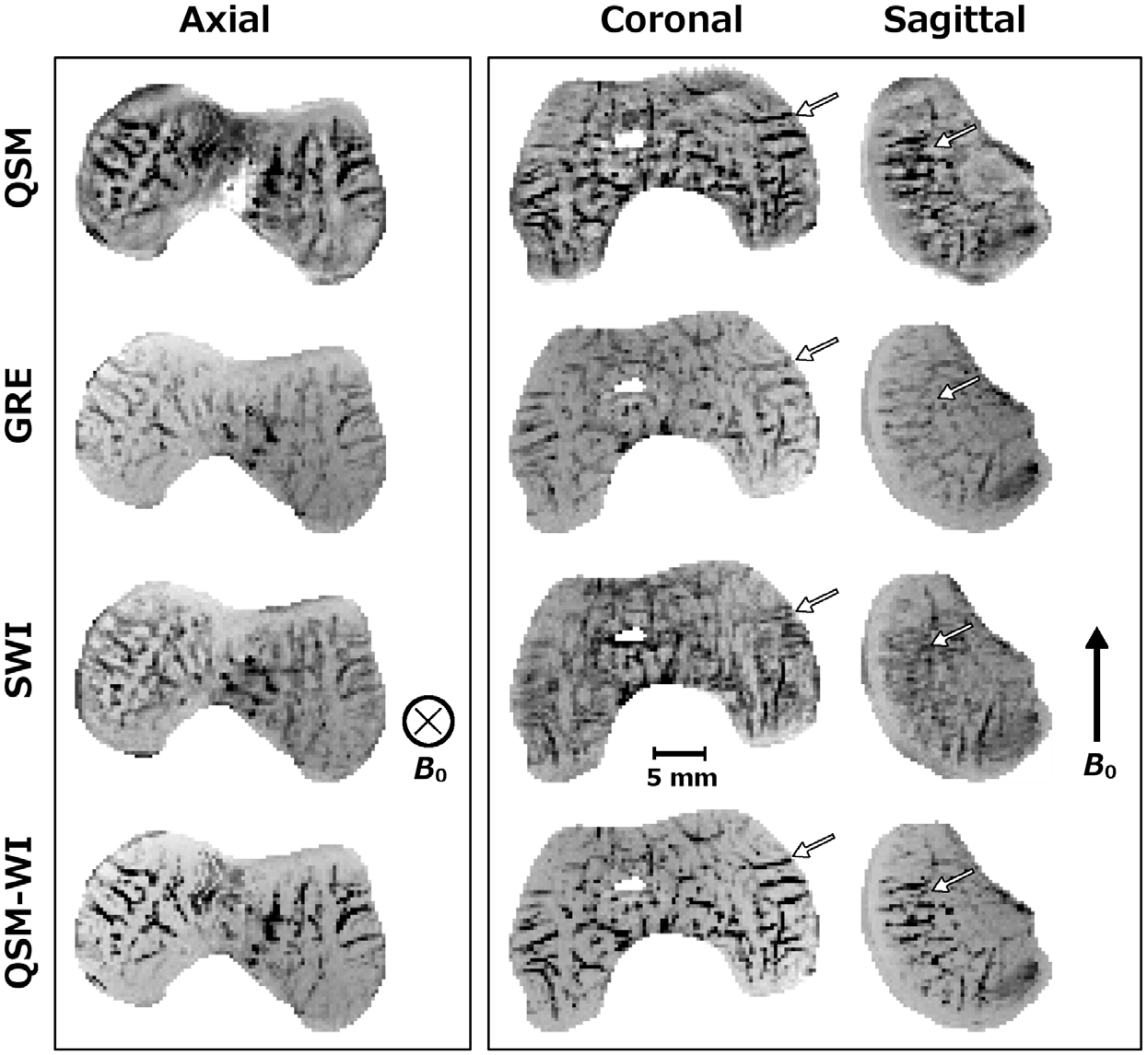
Comparison of QSM, plain GRE, SWI and QSM-WI at 7.0 T *ex vivo*. Comparison of QSM post-processed, plain GRE, SWI and QSM-WI datasets from a 1-month-old human specimen scanned at 7.0 T (TE = 29.06 ms and bandwidth = 60 Hz/pixel) in 3 mm-thick mIPs with QSM contrast inverted to match SWI and GRE. The first pane shows the axial plane, perpendicular to *B*_0_. All four techniques demonstrated closely similar results. In the planes parallel to *B*_0_ (second pane), GRE and QSM demonstrated a closely similar visual appearance; however, the splitting artifact along *B*_0_ was evident in the SWI post-processed data. QSM-WI demonstrated both corrected artifacts and improved visualization of the cartilage canals.

Similar to the results obtained at 9.4 T, the differences between QSM and SWI and QSM-WI post-processing were observed at 7.0 T (Fig 4). In the axial plane, a similar appearance was noted for the 3 mm-thick mIPs of the SWI and QSM post-processed data as well as for plain GRE data (Fig 4, first pane). In the planes parallel to *B*_0_, the splitting artifact was again noted for SWI post-processing, while the QSM, GRE and QSM-WI demonstrated artifact-free visualization (Fig 4, second pane). The sensitivity to the vessels appeared weaker in the un-processed GRE data compared to the QSM or SWI or especially QSM-WI data in the axial plane. Spatial variations in the signal intensity were noted particularly for the unprocessed GRE data and, to a lesser extent, for the SWI data (although these variations were reduced in the minimum intensity projection), whereas the QSM data appeared more uniform in signal intensity. Combination of GRE and QSM-based mask in QSM-WI again provided the clearest visualization.

**Fig 5:**
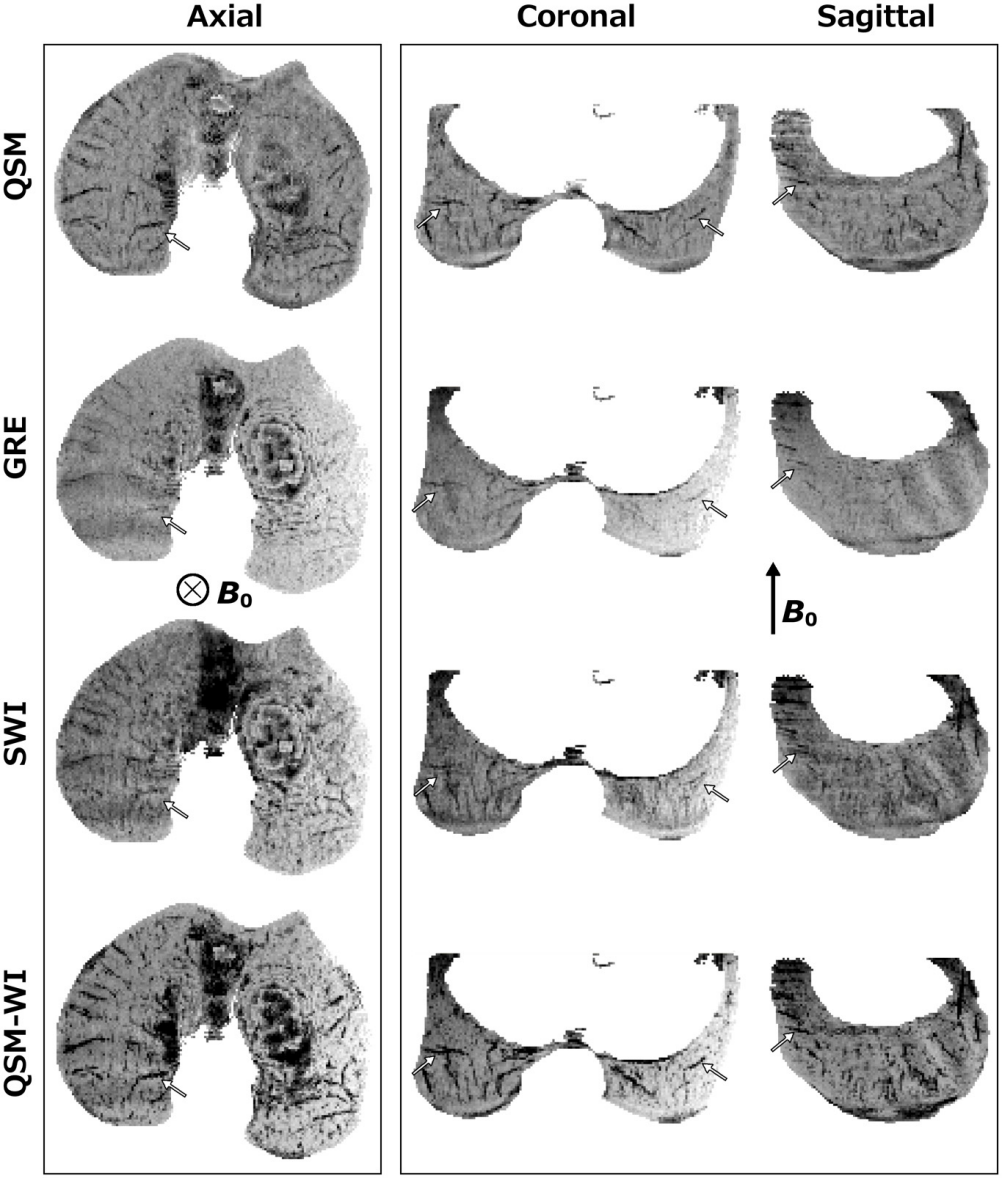
Comparison of QSM, plain GRE, SWI and QSM-WI at 7.0 T *in vivo*. Comparison of QSM, GRE, SWI and QSM-WI of a 3-week-old piglet scanned at 7.0 T *in vivo*. In the first pane, showing an axial plane perpendicular to B0, the datasets appeared visually similar. In the second pane, with views parallel to B0, artifactual splitting of the vessels was observed for the SWI data while both QSM datasets and the unprocessed GRE appeared artifact-free.

The QSM, SWI and QSM-WI post-processing was done for the 3-week-old pig scanned *in vivo* at 7.0 T and compared with unprocessed GRE as well. Results similar to the *ex vivo* cases were obtained. Specifically, in the axial plane perpendicular to *B*_0_, the datasets appeared visually similar (Fig 5, first pane), but, in the planes parallel to *B*_0_, the artifactual splitting of the vessels was observed for the SWI data while in both of the QSM datasets and in the unprocessed GRE the splitting was resolved (Fig 5, second pane). Otherwise, the QSM datasets appeared to maintain the same, if not improved sensitivity to the cartilage canals.

**Fig 6:**
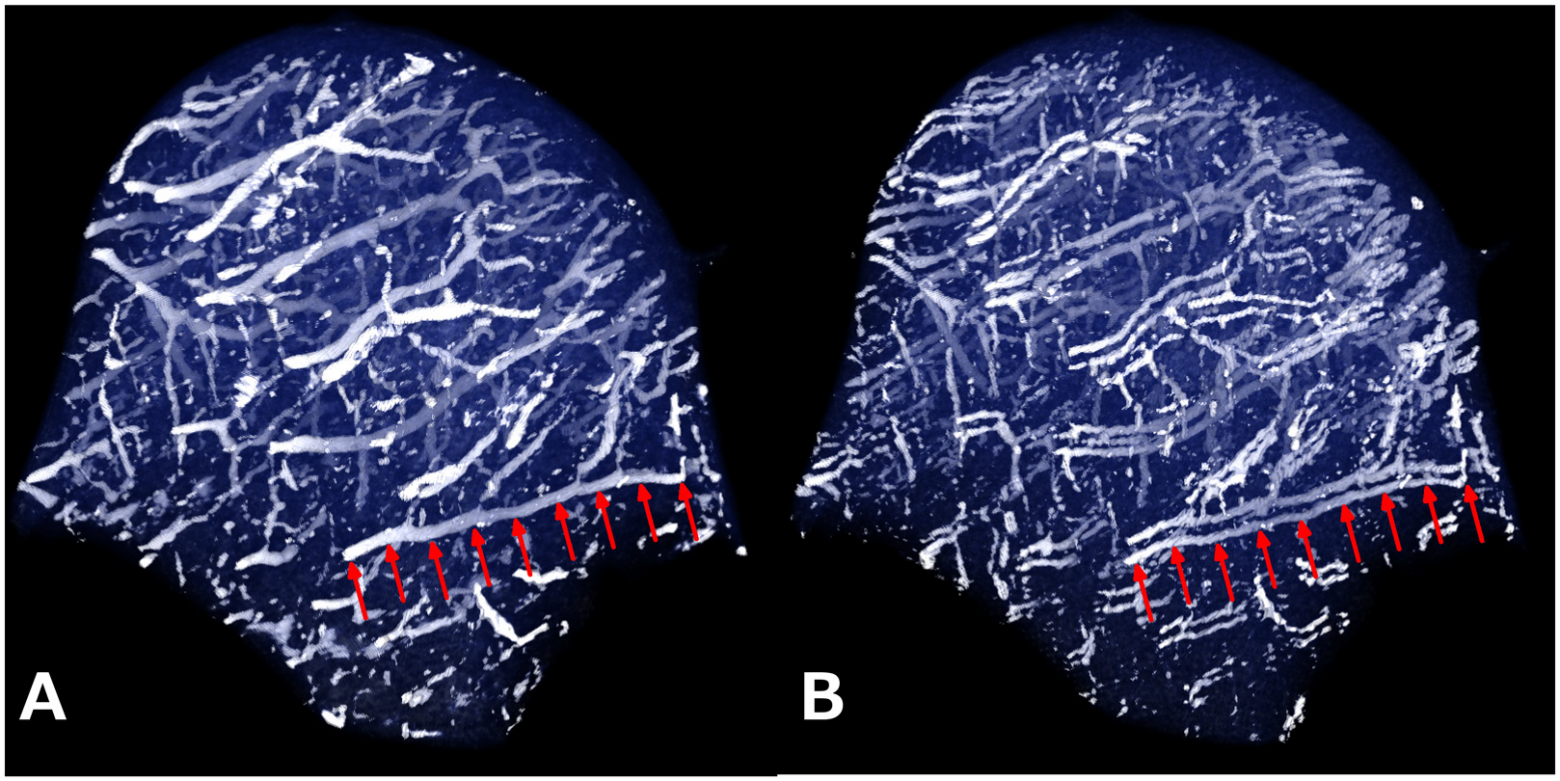
Three-dimensional reconstructions of cartilage canals using SWI and QSM. 3-D reconstructions of the cartilage canals in the medial femoral condyle of an 8-week-old pig scanned at 9.4 T. The QSM processing (A) allowed visualization of the cartilage canals without artifacts. In the SWI post-processed data (B), the splitting artifact was seen. The red arrows point to a matching vessel identified in the two datasets.

Visual evaluation of the three-dimensional reconstructions of the medial femoral condyle of the 8-week-old piglet demonstrated the importance of resolving the artifact to allow detailed assessment of the epiphyseal vasculature (Fig 6). Indeed, the QSM post-processing (Fig 6A) was superior to the SWI post-processing (Fig 6B) by resolving this splitting artifact. A 3-D visualization of the human specimen scanned at 9.4 T is available as a video clip (S1 Video) in the online supporting material, further illustrating the difference between SWI and QSM post-processing methods.

## Discussion

Using the QSM post-processing approach for SWI data substantially improved visualization of the cartilage canals in the distal femoral epiphyseal cartilage in cadaveric human and porcine specimens compared to previous results obtained by SWI post-processing [12, 19]. The discrete dipolar “vessel splitting” artifact, characteristic of the SWI data, was mitigated by the use of QSM post-processing. The combination of GRE and QSM data, i.e. QSM weighted imaging [26, 27], provided the clearest visualization of the cartilage canals in all datasets. While the post-processing method (QSM) itself has been investigated in numerous studies for other tissue types and applications, its use for this particular purpose has not previously been reported and represents a significant advancement in the imaging of cartilage canals. The results using this method are at least as good, if not better, than results obtained by techniques that are limited to *ex vivo* application (perfused-μCT imaging and perfused microscopy-visualization) and which are only applicable to animal studies. Importantly, the non-invasive 3-D visualization of the tissue-inherent contrast potentially allows this technique to be applied *in vivo*.

Processing of the data using SWI resulted in the artificial doubling or splitting of the cartilage canals [12]. This artifact arises from the dipolar accumulation of the phase around a susceptibility inclusion [9, 13] combined with the direct application of a phase mask further enhancing these features [1, 11] in the original SWI post-processing protocol. Depending on whether a negative or positive phase mask is used, the artifact appears as splitting along the *B*_0_-direction or as a ring-like “edge-effect” in the plane perpendicular to *B*_0_. The spatial size of the splitting appears to scale with the scanning resolution and is expected to change with the field strength as well. The appearance further depends on the susceptibility of the inclusion, i.e. the sign of the dipolar phase pattern [9]. In the present case, a negative phase accumulation was evident on the dipole lobes along *B*_0_, indicating positive susceptibility (paramagnetic species). This agrees with the assumption that the signal source (source of the susceptibility difference) is the deoxygenated blood, or remnants of blood in the cartilage canals [11]. Alternatively the cartilage canal itself (separate from the blood within the vessels) may have a magnetic susceptibility value different from the surrounding tissue, enhancing the effect. The susceptibility values of the cartilage canals are not necessarily expected to agree with what is reported for veins, since the cartilage canals contain both veins and arteries. The relative susceptibility of the canals with increasing truncation factor for the k-space filtering kernel approached a range of approximately +0.05 to +0.07 ppm with respect to the surrounding tissue. While these values are approximately comparable to those reported for small veins in brain (0.095 ± 0.006 ppm) [10], susceptibility values for cartilage canals have not been reported previously and further investigations are underway.

The QSM processing of the data eliminated the incorrect localization of the susceptibility source and the artifactual splitting of the vessels along the *B*_0_ direction [12]. The QSM processing in the present study was optimized for visualization purposes: the SHARP truncation factor was set to 0.25 for the *ex vivo* human sample at 9.4 T and to 0.5 for all the other cases, values markedly larger than the value of 0.05 used in previous reports [14, 15]. The k-space filtering truncation was set to the minimum absolute value of the filter, 1.5 (i.e. heavy regularization) [13]. Both of these resulted in reduction of the streaking artifacts at the cost of correctness of the susceptibility values (see also Fig 2). Further reduction of the truncation value below the smallest absolute values of the kernel did not improve the relative intensity of the streaking artifacts. An alternative approach for reducing the artifacts could have been setting the truncated values to zero [17]. For quantitative susceptibility analysis, a different choice of the kernel truncation, such as the value at the elbow of the curves in Fig 2, could be more appropriate, or a correction scheme as presented by Schweser et al could be utilized [29]; however, improvement of the signal localization over SWI processing was sought and achieved. This interdependence between the artifacts and accuracy of the quantitative susceptibility values represents a major drawback of QSM. For the 3-D evaluation of the vasculature, splitting along one dimension hampers visual assessment of the integrity and connectivity of the vasculature (see also the video clip (S1 Video) showing rotation of the 3-D reconstruction in the online supplemental material). An additional benefit of the QSM data is that it is quantitative and provides numerical values for the susceptibility differences between the tissues. In the present case, the cartilage canals had a higher susceptibility compared to the surrounding tissue (i.e. the canals appear paramagnetic). A potential future quantitative application could be assessing the oxygenation of the tissue and the vessels [30].

For the purpose of visualization of the vasculature, the QSM approach could be simplified. Specifically, the source phase data for the truncated k-space QSM filtering could be produced using homodyne filtering [13, 24] instead of using the more complicated Laplacian unwrapping and SHARP filtering [14]. Conversely, the unwrapped phase data obtained via the more complicated approach could be used for SWI post-processing. In terms of computational cost, the QSM post-processing using the approach of Laplacian unwrapping followed by SHARP filtering and truncated k-space filtering took approximately 43 s on a standard laptop using a quad-core CPU, whereas using homodyne filtering and the same truncated k-space filtering took approximately 16 s on the same computer, using the same dataset (a matrix size of 384 cubed).

Clear visualization of randomly oriented cartilage canals [31], some of which are less than 100 μm in diameter [12, 21], requires high or ultra-high resolution MRI. This may partially limit the application at lower field strengths, such as 1.5 T, as the SNR drop will influence the achievable resolution and image quality. While the cartilage canals were visualized at both 7.0 T and 9.4 T, the lower quality at 7.0 T is largely due to the suboptimal (too large) coil that was available at 7.0 T [12]. Even with these limitations, the results obtained at 7.0 T confirmed the observation that the QSM post-processing approach eliminated the visualization artifacts and provided improvement over SWI. The SWI data demonstrated similar clear contrast between the cartilage canals and the surrounding tissue only when data were acquired in the axial plane, which hides the dipolar splitting artifact. Similar to the QSM data, the canals were also visualized without notable artifacts in the plain, unprocessed (excluding the minimum intensity projection) GRE data. This suggested that a combination of GRE and QSM could further improve visualization of the cartilage canals. “Enhanced SWI”, QSM-WI, was generated for the investigated cases by using the susceptibility map as an enhancing mask, in a similar manner as the phase mask is used in SWI (last row in Figs 3–5). Here, QSM-WI was applied to test whether visualization improvement was achievable; more detailed approaches for similar processing are provided in earlier publications, which referred to the method as susceptibility map weighted imaging (SMWI) [26] or as true susceptibility weighted imaging (tSWI) [27]. The QSM-WI data appeared to be qualitatively superior to the QSM-only visualization and might represent the ideal processing pathway for visualization of cartilage canals.

At ultra-high magnetic fields, the wavelength of resonant radio waves is not long relative to body dimensions; this results in *B*_1_^+^ inhomogeneity and thus variations in signal intensity in the targeted tissue. At 7.0 T, *B*_1_^+^ shimming was available and was used, generally improving the spatial homogeneity of the signal within the targeted tissue. At 9.4 T, the quadrature coil used did not allow this option. However, very good overall homogeneity of the signal was observed in the 9.4 T scans. Although the specimen that was scanned at 7.0 T was small (the largest width of the distal femur was approximately 3 cm), spatial variation of the signal due to variations in *B*_1_^+^ was noted. This spatial variation was reflected in both the SWI processed and the plain GRE data, and can be appreciated in Fig 4 and Fig 5. In the QSM data, derived from the background-corrected phase, spatial variation in the intensity level was not evident. To achieve similar homogeneity in the SWI or GRE data, separate spatial intensity correction could be utilized. For the 3-D visualization of the cartilage canals by volume rendering, spatial homogeneity of the tissue matrix is essential. Although some spatial variation of the signal and generally lower resolution was noted at 7.0 T as compared to the acquisitions at 9.4 T, the benefits of QSM are also demonstrated at this lower field strength, which is closer to that used clinically.

The processing pathways chosen in the present study are examples of the multiple possible combinations of procedures employed for QSM (or SWI). The segmentation mask and its quality substantially affected the final visualization, especially the 3-D reconstruction: low-signal regions included in the segmentation mask could cause streaking artifacts that generate noise and fluctuations in the final QSM images. Thus, one key element in the QSM processing and visualization is the segmentation of the desired region of interest. Due to multiple tissue components exhibiting the same or similar signal levels, simple thresholding generally does not perform well, necessitating laborious manual segmentation. Several segmentation methods, including manual segmentation, were used in the present study. Furthermore, both methods presented here (SWI and QSM) benefit from higher field strengths. In addition to the suboptimal coil, the difference in the field strength contributes to the somewhat poorer SNR and contrast of the vessels at 7.0 T compared with the results obtained at 9.4 T. As noted in the earlier report utilizing only SWI, visualization of the cartilage canals was possible at 3.0 T, albeit at lower SNR [12]. Thus a challenge for clinical application, in addition to segmentation, is the achievable SNR. Therefore, a dedicated pediatric knee or extremity coil is strongly recommended. The QSM post-processing, due to its ill-posed inverse nature, tends to propagate and amplify k-space noise to the derived images; thus, the applicability of the method needs to be demonstrated at clinically relevant field strength to prove its utility for routine clinical applications. The [current] limitations of QSM processing include the requirement of transferring data for off-line processing, as there currently are no scanner-implemented solutions available. Even with off-line processing, interpretation of the vascular findings relies on subjective visual evaluation. The present results pave the way for implementing an automated and quantitative analysis of the vasculature, which would markedly improve the ability to interpret the results. Another possible limitation of the study is that the specimens underwent a freeze-thaw cycle before scanning; however, the cartilage canals were clearly visualized and in the previous study including histology, no effects of freezing on the integrity or structure of cartilage canals was noted [12, 19]. Finally, since cartilage canals are no longer present after the age of approximately 10 years in human beings, appropriate MR imaging protocols for children will need to be developed.

The initial SWI studies were the first studies to demonstrate the potential utility of MR imaging of cartilage canals, including its *in vivo* application [12, 19, 32]. The present work improves the method further by demonstrating resolution of the “splitting artifact” observed in SWI. Potential future applications include *in vivo* imaging of cartilage canals in animal models of human disease as well as *in vivo* imaging in humans. Further work is needed to utilize the method for noninvasive assessment of blood oxygenation levels of epiphyseal cartilage canal vessels *in vivo*, which would not only be useful for the assessment of normal epiphyseal development but also its alterations in diseases such as osteochondrosis and Legg Calve Perthes disease, which have not been studied *in vivo* mainly due to a previous lack of noninvasive investigational tools [19].

## Conclusions

In the present work, improvement of the *ex vivo* and *in vivo* visualization of epiphyseal cartilage canals was demonstrated with QSM compared to the previously reported SWI method. The main limitation of SWI post-processing is the artifactual splitting of small susceptibility inclusions, such as the cartilage canals, along the *B*_0_ direction due to the dipolar phase accumulation. The QSM post-processing not only reveals the underlying susceptibility distribution, but also resolves its spatial localization and thus enables precise visualization. GRE imaging optimized for SWI, but utilizing QSM post-processing provides highly accurate, reproducible 3-D visualizations of the vascular network in epiphyseal cartilage and will likely open new avenues to investigate the etiology of diseases associated with abnormalities of skeletal maturation, many of which appear to be related to defects in vascular supply to epiphyseal cartilage.

## Supporting Information

S1 Video Comparison between 3-D reconstructions with SWI and QSM post-processing. A video clip demonstrating the difference between the 3-D reconstructions of the cartilage canals of the distal femur of a 1-month-old human cadaver as produced by SWI and by the truncated k-space filtering QSM approach.
(MP4)
